# SIESTA: a quick interprofessional learning activity fostering collaboration and communication between paediatric nursing trainees and medical students

**DOI:** 10.1186/s12909-021-02880-9

**Published:** 2021-09-06

**Authors:** Sebastian Friedrich, Christine Straub, Sebastian Felix Nepomuk Bode, Andrea Heinzmann

**Affiliations:** 1 Department of General Pediatrics, Adolescent Medicine and Neonatology, Medical Centre, Faculty of Medicine, University of Freiburg, Freiburg, Germany; 2 Department of Pediatrics and Adolescent Medicine, Ulm University Medical Center, Ulm, Germany

**Keywords:** Interprofessional education, Interprofessional training ward, Peer teaching, Paediatrics, Time limitation, Interprofessional communication, Interprofessional collaboration

## Abstract

**Background:**

Interprofessional education has emerged as a key concept in education of health professionals over the last 20 years. Positive effects of interprofessional education have been shown, but it has proved to be more time-consuming than traditional teaching methods. We therefore developed a 30-minute interprofessional learning activity, using peer-teaching methods. We were interested in effects on and ways of interprofessional learning, including conditions and resources that make it successful despite limited time.

**Methods:**

Speed InterprofESsional Peer Teaching PaediAtric (SIESTA) was developed in the context of an interprofessional training ward. 20 paediatric nursing trainees and 20 medical students were enrolled in the study. Two students from each profession participated in a total of four SIESTA sessions each, supervised by registered paediatric nurses and paediatricians. We used a mixed-methods approach of quantitative and qualitative data (questionnaires, semi-guided focus group interviews) to evaluate self-perceived interprofessional competencies, interprofessional learning gains and ways of interprofessional learning.

**Results:**

Questionnaires were obtained from all participants (*n* = 40) and *n* = 26 took part in the group interviews. Participants from both professions reported an increase in self-perceived understanding of interprofessional roles and tasks. Communication and cooperation emerged as important aspects. The workplace-based nature of SIESTA promoted interprofessional learning, while peer teaching fostered a safe learning environment. Regarding time constraints participants suggested thorough preparation and structuring by facilitators as a solution.

**Conclusions:**

Our short interprofessional peer teaching activity showed promising results. Participants reported enhanced interprofessional competencies and provided suggestions for successful learning in limited time. Further studies should include an objective assessment of the interprofessional learning progress. The SIESTA concept can be easily adapted to other medical fields, providing interprofessional learning opportunities for many more health care professionals to come.

**Supplementary Information:**

The online version contains supplementary material available at 10.1186/s12909-021-02880-9.

## Background

Health care professionals are facing increasing workload and time pressure [1–3]. In order to be able to complete more tasks in less time while maintaining highest quality of treatment and care, communication between team members is essential [4–6].

Efficient communication among health care professionals has been shown to promote both feedback culture and patient safety [7]. While efficient communication among members of one healthcare profession is of great value, it may not be enough.

Interprofessional communication and interprofessional collaboration (IPC) have been discussed for 50 years, but only came to a broader public’s attention over the last two decades [8, 9]. Both have been shown to improve satisfaction among staff and patients [10]. Reported positive effects include patients feeling better informed, earlier discharge of patients and cost-effectiveness [11, 12]. However, more objective patient outcome parameters have not yet been reported, mainly due to methodological constraints [13–15].

Implementing an interprofessional (IP) approach to health care is a long-term process that involves a change of culture on many levels. According to the World Health Organization, interprofessional education (IPE) is defined as students from two or more professions learning from, with and about each other, enabling effective collaboration and improving health outcomes [16]. The need for interprofessional education (IPE) as a prerequisite for interprofessional collaboration is summarized by the question “How can they work together if they do not learn together?” [17]. To answer this question, projects addressing IPE in healthcare have been implemented around the world [18, 19]. However, the optimal timing to introduce IPE in the medical and nursing curriculum is still subject of ongoing research. Results hint to an early introduction of IPE being favourable. There is still uncertainty whether continuous IPL (immersion) or repeated short IP activities (exposure) lead to better results. [20, 21].

A success story of IPE is the implementation of IP training wards. IP training wards are a key factor for providing workplace-based interprofessional education to learners from different backgrounds [10]. Moreover, focusing on IPE is encouraged by health education institutions in many countries such as Canada, the United States, Australia, Sweden, Switzerland and Germany [9, 20, 22–25].

At our faculty, an interprofessional training ward in the setting of a general paediatric ward (IPAPAED) has been established in 2017. A short summary is provided in the methods section. To complement hands-on learning during direct patient care on the ward, we developed an additional learning activity, Speed InterprofESsional Peer Teaching PaediAtric (SIESTA). The concept of SIESTA sessions was based on several considerations:

Firstly, time limitations are a part of daily life in healthcare [3] and therefore SIESTA was planned as a 30 min session.

Secondly, peer teaching has been shown to be well accepted by interprofessional team members and promote professional conduct, understanding and feedback culture [26–28]. Topping defines peer teachers as people from similar social groupings who are not professional teachers helping each other to learn and learning themselves by teaching [29]. We wanted to enable participants to emphasize the interprofessional aspect of learning activities and limit complexity on an academic level. Hence, we decided to limit patient selection to frequent paediatric clinical problems, e.g. acute bronchitis, gastroenteritis, and minor blunt head trauma.

Thirdly, principles of self-directed learning were adhered to as participants agreed on a specific topic arising from a patients’ clinical presentation and agreed on learning goals for their SIESTA session. Self-directed learning puts learners in a position of motivation, responsibility and control over the learning process [30, 31]. As self-directed learning is becoming more and more important in the context of life-long learning and continuing medical education, we decided to use it as a foundation for the SIESTA concept [32, 33]. Additionally, as patient-centred care was one of the core principles behind the IPAPAED training ward, it seemed natural to centre the SIESTA learning session around a patient’s clinical problem.

We defined learning goals for SIESTA sessions as follows:

By completing SIESTA, participants should be able to….

- …understand the other professions’ roles and appreciate their expertise.
- …explain their own point of view as a member of an interprofessional team and discuss questions with the other profession.
- …select contents they consider relevant regarding patient care.

Implementing SIESTA, we were interested to see whether successful interprofessional learning (IPL) was possible in a time-limited and peer-led format. Different studies have evaluated the effects of time-limited IPL interventions in the past [21, 34]. However, to our knowledge this is the first study to evaluate the effects of a time-limited IPL activity combined with a peer-teaching approach. Combining both aspects might contribute to an easier implementation of IPL activities by saving time and faculty resources. Ultimately, SIESTA could be adapted to different scenarios, based on thorough evaluation of the initial concept.

To ensure a thorough evaluation, we used a mixed-methods approach consisting of both quantitative and qualitative data. Qualitative data were used to validate findings from quantitative data. We wanted to explore how participants reflected on their own learning regarding interprofessional communication and collaboration. Specifically, we aimed to identify factors that made participants learn from, with and about each other during SIESTA sessions.

We therefore addressed the following research questions:

1. Does our learning activity promote interprofessional communication skills and knowledge?
2. In which way do paediatric nursing trainees and medical students learn from, with and about each other during SIESTA?
3. What is needed to foster interprofessional learning for paediatric nursing trainees and medical students in this time-limited format and how can facilitators from both professions support the learning process?

## Methods

### Context of the Study and Participants

**Fig. 1:**
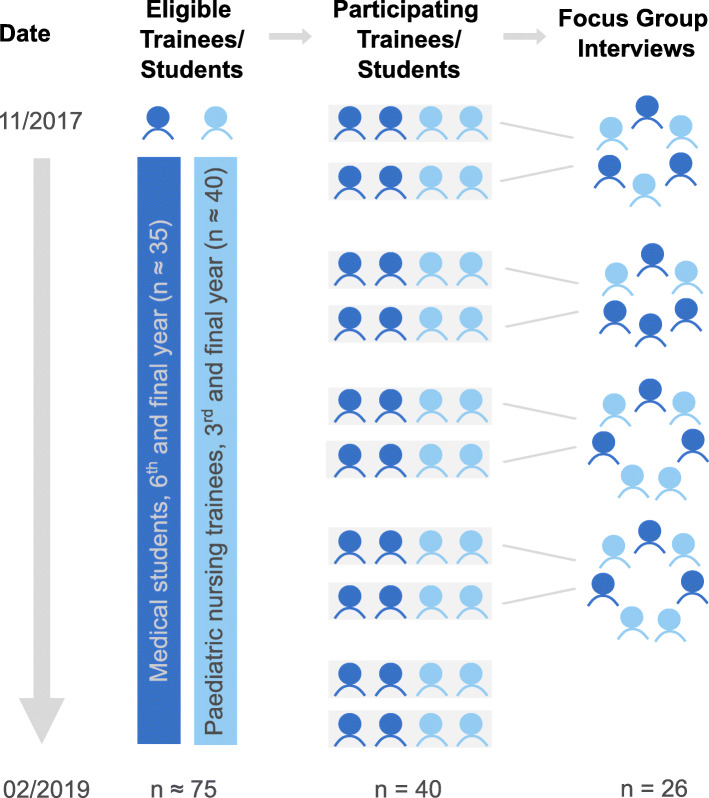
Trial flow. About 35 medical students (MS) and 40 paediatric nursing trainees (NT) were eligible for participation between November 2017 and February 2019. 20 MS and 20 NT participated in the study, forming 10 interprofessional teams. Focus group interviews were conducted with 13 MS and 13 NT, interviewing participants from two different interprofessional teams together

SIESTA was implemented in the framework of IPAPAED. The characteristics of this training ward have been published previously [35, 36]. Paediatric nursing trainees (NT) in the final year of a three year training and medical students (MS) in the 6th and final year of studies (two years pre-clinical, 4 years clinical) participated in the project and hence in the SIESTA study. Together, they spent two weeks on IPAPAED as an interprofessional team. Four SIESTA sessions were held during the two-week period. NT were assigned as participants according to their schedules and organisational circumstances defined by the nursing school. MS were assigned to the project if they were on a rotation in the paediatrics department at the time of the study. In some cases, the number of MS interested in the project exceeded the available places, so participants were randomly assigned. Figure 1 illustrates the selection of participants and formation of teams. For each team, experienced medical and nurse facilitators trained in interprofessional teaching provided supervision. At the beginning of each placement, participants were introduced to interprofessional collaboration and communication.

### SIESTA concept

**Fig. 2:**
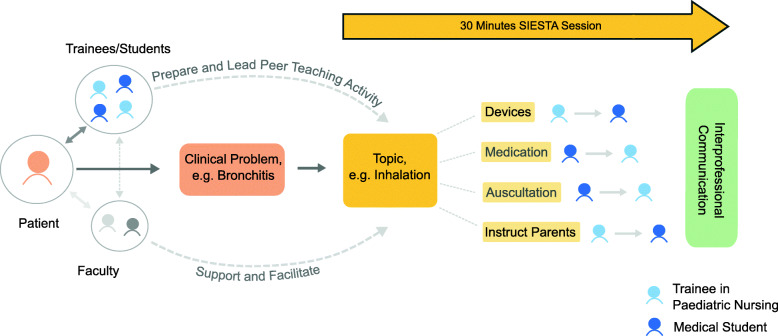
Sample SIESTA session. An interprofessional team of two paediatric nursing trainees and two medical students provides care for a patient. Based on a patient’s clinical problem they chose a topic for their SIESTA peer teaching session. Interprofessional facilitators provide support when needed. SIESTA: Speed InterprofESsional Peer Teaching PaediAtric

The day before the SIESTA session, facilitators asked the interprofessional team to choose a topic for SIESTA, related to a patient’s clinical problem (e.g. inhalation related to bronchitis). The team agreed on a topic and together they prepared the learning activity. Team members from both professions prepared profession-specific aspects of the topic (see Fig. 2). During the 30 min SIESTA session, they demonstrated and explained these aspects to their peers from the other profession. During peer teaching, participants applied communication and collaboration.

Interprofessional facilitators (a registered nurse educator and a physician known to the team) provided preparatory material, such as standards of care and standard operating procedures as well as materials (e.g. inhalation devices). They assisted during preparation, when necessary and were present during the SIESTA session. Thus, interprofessional collaboration by teaching staff provided an excellent opportunity for role-modelling [37]. One sample SIESTA session is illustrated in Fig. 2.

### Survey and group discussions

The study was designed as a single group post-test only study. The number of participants was limited by the number of students on IPAPAED. Previous studies have demonstrated that for IPL, social interaction between participants from different professions is crucial [38]. Using a single-method approach in IPL can limit findings substantially [21]. We therefore opted for a mixed-methods approach, combining quantitative and qualitative data. Qualitative data were used to validate findings from quantitative data, aiming at methodological triangulation [39]. Quantitative data were collected using questionnaires. The questionnaire was developed based on previously published and validated questionnaires and discussed within the research team before use [40–42]. A five-point Likert-scale was used for grading. To address research question 1, we included items about interprofessional collaboration and communication (items 1 and 2 and 3). Regarding research question 2, we asked for self-perceived learning gains (item 4 and 5). For research question 3, we asked for participants’ attitudes regarding duration (item 6). The items included in the questionnaire are provided in Table 1:

**Table 1:** Items from SIESTA questionnaire

1	Learning with and from the other profession during SIESTA contributed to a better understanding of the other profession’s tasks in patient care.	strongly agree	agree	undecided	disagree	strongly disagree
2	Learning with and from the other profession during SIESTA made it more likely for me to seek advice from the other profession regarding patient care.	strongly agree	agree	undecided	disagree	strongly disagree
3	Please rate the interprofessional exchange among the SIESTA participants.	excellent	good	fair	poor	very poor
4	The content discussed during SIESTA was highly relevant (e.g. for exams, professional work etc.).	strongly agree	agree	undecided	disagree	strongly disagree
5	Please rate your learning gains through the SIESTA course.	very high	high	medium	low	very low
6	Please rate the duration of SIESTA (30 min).	far too long	too long	just right	too short	far too short

The full questionnaire is provided as additional file 1.

Questionnaires also included open-ended questions to collect qualitative data. Participants were invited to indicate which further material or equipment they would have liked for SIESTA; what they liked regarding SIESTA and what could be improved in their opinion. As a second approach to qualitative data collection, we conducted semi-structured focus group interviews.

### Data collection and analysis

Data were collected from 40 SIESTA participants between November 2017 and February 2019. Questionnaires were handed out to participants at the end of their two-week placement. Quantitative data were analysed with Graph Pad Prism version 8.4.3 (GraphPad Software, La Jolla, California, USA). Likert scale data were treated as ordinal data; therefore no mean or standard deviations are reported. The Mann-Whitney-U-test was used to identify differences between paediatric nursing trainees and medical students. Significance was defined with an alpha level of 0.05.

Semi-structured focus group interviews were conducted at the end of two consecutive IPAPAED placements. Part of the interview was dedicated specifically to SIESTA sessions. A total of four group discussions was held, with two groups of six and two groups of seven participants. Two interprofessional teams each were interviewed together (see Fig. 1). Discussing with two independent groups enabled participants to bring up new aspects that might have been missed using standardised questionnaires only [43]. Group discussions were conducted by co-author CS and audio-taped. Duration of discussions was between 40 and 60 min. Recordings were then transcribed verbatim by an independent facility [44]. For analysis of qualitative data, we performed deductive and inductive category assignment [45]. For each research question, two or three categories were assigned, with paraphrases describing each category. Categories were determined based on the „Core Competencies for Interprofessional Collaborative Practice“ and assigned to our research questions [16]. Additionally, categories were derived inductively from the focus group interviews to answer research question 3 [45].Transcripts were coded and quotes were assigned to the specific categories. Single quotes were given a unique identifier, composed of the number of group discussion and lines in the specific transcript (e.g. 1 / 280–282). Additionally, answers to open-ended questions from SIESTA questionnaires were coded using the same approach. Answers from questionnaires were assigned a number, according to pooled answers from one IPAPAED placement (e.g. 1, 2, 3). Coding and category assignment were performed independently by two members of the research team, CS and SF. Areas of disagreement were discussed until consensus was reached. The complete coding scheme, including sample quotes and category assignment, is provided in Table 2. Sample quotes were translated from German into English in a two-step approach: First, quotes were translated verbatim by authors CS and SF and then proofread by a native English speaker to improve understanding by the reader.

### Ethics

SIESTA sessions were held in the framework of IPAPAED placements. The latter were approved by the University of Freiburg ethics committee (permit no. 561/17). For questionnaires, all participants gave written informed consent. For group interviews, all participants equally gave written informed consent prior to audio taping. All data were pseudonymised. Electronic data were stored on a password-protected local server. Paper-based questionnaires were stored in a locked container inside a locked office. Data will be destroyed after 10 years. Only members of the research team had access to the data.

**Table 2:** Systematic coding scheme for research questions (deductive category assignment)

Research Question	Category and Description	Sample quotes from group discussions (d) and text comments (c)
**1. Does our learning activity promote interprofessional communication skills and knowledge?**	1.1 communication Open verbal exchange within the interprofessional team, sharing and enhancing knowledge and expertise	„structured discussion of this case … considering the respective nursing and medical perspective“(c, 1) „it was also nice to complement each other and add criticism and suggestions“ (c, 3)
	1.2 mutual cooperation - Cooperation on a professional level and arrangements between professions - better understanding about the other professions‘ perspective	“Choosing topics together depending on current clinical problems“ (c, 2) „Insight into the other professions‘ perspective“ (c, 2)
	1.3 professional roles Knowledge about duties and tasks of both professions	„Improved my understanding of the nurses‘ tasks“ (c,2) „Gain insight into what physicians learn, better understanding“ (c, 2)
**2. In which way do trainees in paediatric nursing and medical students learn from, with and about each other?**	2.1 Interprofessional learning Interprofessional learning in a work-place based context	If possible, topics with a practical side to it, material…“ (c, 1) „Good level between terminology and simple explanations“ (c, 2)
	2.2 Peer Learning activities Interprofessional and profession-specific learning on equal terms in a good learning atmosphere	„You feel as a team, discuss everything and explain things to each other“ c, (2)
**3. What is needed to foster interprofessional learning for paediatric nursing trainees and medical students in this time-limited format and how can facilitators from both professions support the learning process?**	3.1 Conditions Select and prepare relevant topics using adequate methods und discuss them in an appropriate time format	„Clinical problems that were relevant to our current patients“ (c, 2)
	3.2 Resources Providing both material and support, structuring when needed, creating a stimulating learning atmosphere.	„To think about a concept beforehand: all right, these points should all be addressed during SIESTA“ (d, 3, 340 f.) „To have a specialist from both professions“ (c, 2)

## Results

### Participation and return

Questionnaires were obtained from all 40 participants (return 100 %, NT n = 20, MS n = 20). Thirteen NT and 13 MS participated in the semi-structured focus group interviews. Data for all answers given are provided in additional file 2.

### Promotion of interprofessional communication and knowledge

**Fig. 3:**
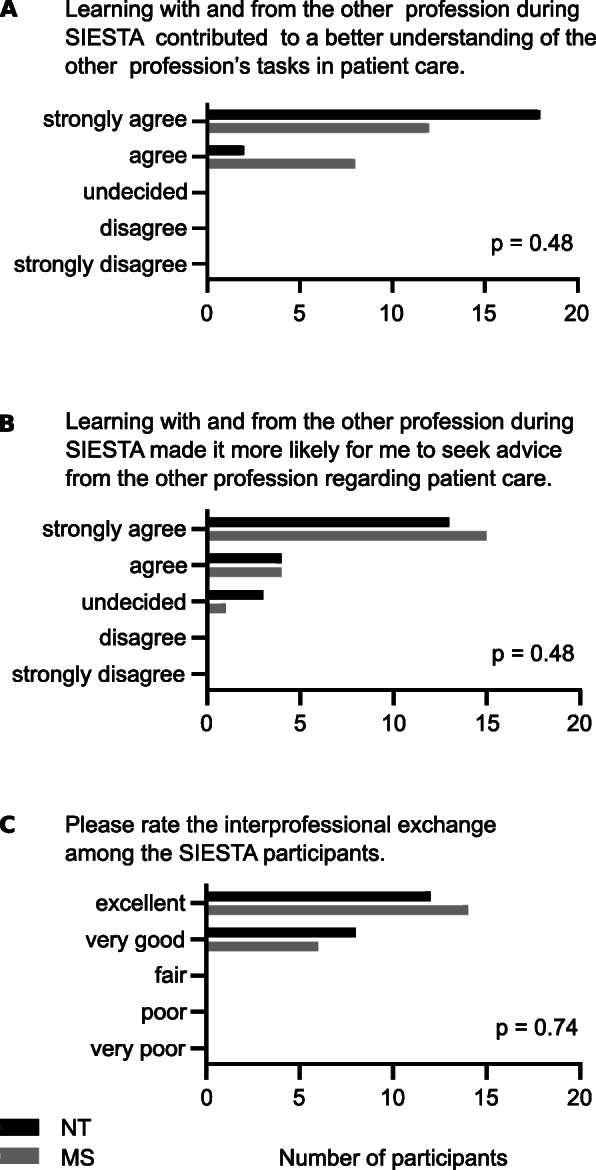
Interprofessional collaboration and communication. **A**: Number of answers given for question 1. **B**: Number of answers given for question 2. **C**: Number of answers given for question 3. NT = paediatric nursing trainee, MS = medical student. *P*-values between professions, using Mann-Whitney-U-test.

SIESTA sessions promoted self-perceived understanding of interprofessional roles and tasks. All participants strongly agreed or agreed that learning with and from the other profession during SIESTA contributed to a better understanding of the other profession’s tasks in patient care (see Fig. 3 A). No significant difference was detected between professions (*p* = .48). Likewise, interprofessional communication was facilitated, as 85 % (n = 17) of NT and 90 % (n = 18) of MS strongly agreed or agreed to the following statement: “learning with and from the other profession during SIESTA made it more likely for me to seek advice from the other profession regarding patient care” (see Fig. 3B). Again, no significant difference was detected between professions (*p* = .48). The interprofessional exchange among SIESTA participants was rated as “excellent” or “good” by all participants (*p* = .74) (see Fig. 3 C).

Interprofessional communication emerged as a key aspect in group discussions and text comments. One participant highlighted the awareness for differences existing between professions: “… because everything you’ve just talked about briefly will be re-discussed in more detail. And then you realize what kind of differences actually exist. Which different approaches can be used? And then you go through it again and discuss what might be useful.“ (4 / 309–312). Using a patient case as a basis for SIESTA was mentioned as a valuable experience: “structured discussion of this case […] considering the respective nursing and medical perspective [2]“. Uniting around a patient’s problem also fostered cooperation, as it was seen as “a good opportunity to bring together the nursing and the medical team” (1 / 526f). Not only were interprofessional communication and cooperation enhanced, understanding each other’s professional roles was mentioned, too. Medical students “improved [their] understanding of the nurses‘ tasks“ [2] and paediatric nursing trainees were given a better idea about “what physicians learn” [2]. Importantly, exposing the gap between the own and the other profession’s knowledge changed participants’ attitudes regarding interprofessional collaboration: “I think it really makes you value the other profession, especially from a physician’s perspective. You think you know it all after six years and then you come here and see, oh, others will approach the exact same problem in a different way and they know a whole lot of things that you haven’t even heard about.“ (4 / 324–329).

### IPL strategies

**Fig. 4:**
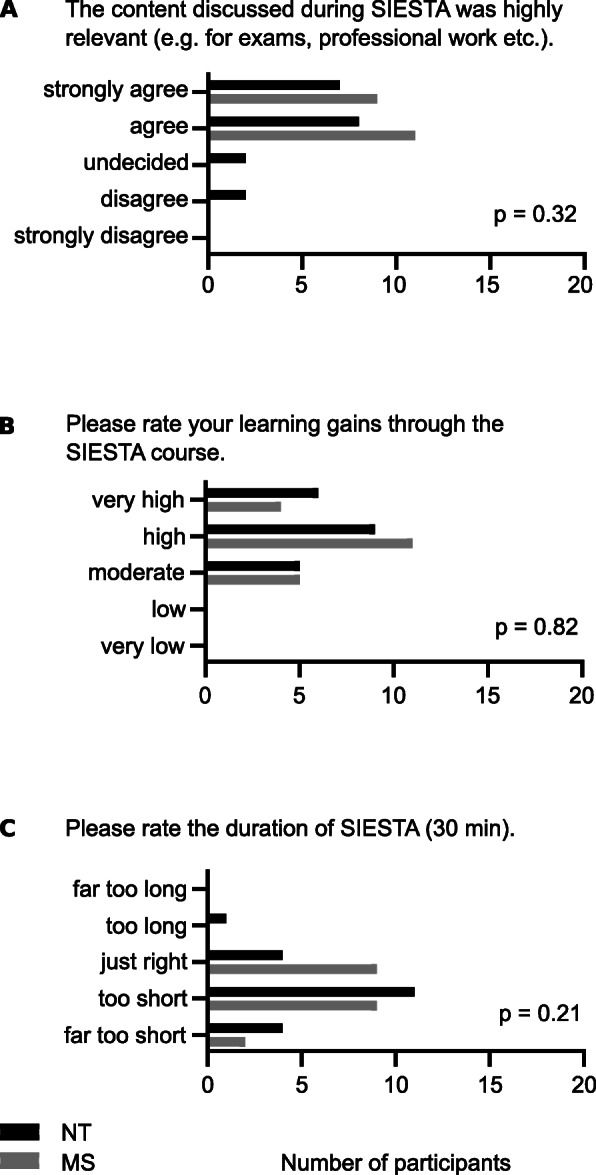
Content, learning gains and duration. **A**: Number of answers given for question 4. **B**: Number of answers given for question 5. **C**: Number of answers given for question 6. NT = paediatric nursing trainee, MS = medical student. *P*-values between professions, using Mann-Whitney-U-test.

Looking more specifically at ways of IPL revealed different important aspects. All MS strongly agreed or agreed that content discussed during SIESTA sessions was highly relevant to them. While 78 % (n = 15) of NT shared this view, 11 % (n = 2) were either undecided or disagreed (*p* = .32) (see Fig. 4 A). Seventy-five percent (n = 15) of NT and MS rated their learning gains as “very high” or “high” (see Fig. 4B).

Two core aspects emerged from qualitative data analysis. Firstly, the workplace-based aspect of IPL during SIESTA was highlighted. As one participant stated, learning was seen „more in the sense of doing ultrasound together, taking blood from each other, things like that. We always had so many things that we wanted to show to each other“ (1 / 600–602). In addition, choosing a common topic gave both NT and MS the chance to “sit down together in the morning and discuss a little and prepare yourself“ (3 / 225–227). Secondly, peer-learning activities promoted learning at eye level in a safe learning environment, despite different professional backgrounds. Participants felt free to ”openly discuss in a familiar environment and ask […] questions” [2] and find out about “the other profession’s expertise („How do you do that exactly?“) [2]. This learning atmosphere was not restricted to SIESTA sessions, but SIESTA topics gave rise to further interprofessional exchange: “I really had the feeling that we almost talked more about the topics during our shift and SIESTA was a kind of a short summary“ (3 / 214–217). Learning from, with and about each other was regarded as bringing together “…like two parts of a whole“ (4 / 315–322).

### Enabling IPL in a time-limited format

Participants highly appreciated SIESTA. Even though they wished for longer sessions (Fig. 4 C), they highlighted that the short time was long enough for a beneficial IPL activity, given that their own preparation was appropriate.

 Many participants provided suggestions and comments: “What did you like regarding SIESTA?” was answered by 39 participants (NT n = 20, MS n = 19), “What could be improved?” was answered by 30 participants (NT n = 15, MS n = 15), “Which further material or equipment would you have liked for SIESTA?” was answered by 29 participants (NT n = 14, MS n = 15), and “Additional comments” were given by 17 participants (NT n = 8, MS n = 9). Topics of the additional comments included thanking the responsible teaching staff for organizing the project, giving suggestions as to organisational improvements and general reflections on what students had learned.

Selection of topics and preparation played an important role. Learning occurred around “clinical problems that were relevant to our current patients“ [2]. Depending on additional work, preparation was facilitated: “I found that preparing for the SIESTA-topics really went well. We usually had cases that we had already seen, like febrile seizures or bronchitis. And then you read and look up things, but that was pretty much it.“ (3 / 282–287) When time was scarce, support by interprofessional facilitators became more important: “Because we were well supported by our supervisors, you did have the time [to prepare for SIESTA].“ (3 / 253–255). Facilitators played an important role, not only to compensate for a lack of time: They were also regarded as an important resource in guiding learning activities. For example, when discussions became too long, it was seen as a task of the “responsible supervisor to say: okay, now we really have to move on from here“ (4 / 323–325). Moreover, input from “experts contributing different perspectives and knowledge” [2] and “experts from both professions, so correctness of things discussed was guaranteed“ [2], was appreciated. Additional support was given by providing material, such as „devices for inhalation“ [2] and on some occasions wished for, such as “have some of the things written down, so you could come back to it.“ (1 / 619–622) Overall, the impression was that „… supervision is really good.“ [4].

## Discussion

Interprofessional education is increasingly being integrated into health care curricula around the world [17, 32, 33]. Preparing health care professionals for successful IPC is a prerequisite for a better, integrative health care and treatment [19]. However, busy schedules for both learners and facilitators can be a constraint to effective implementation of IPE [10]. SIESTA was designed as a short IPL activity using peer teaching as a core method. The data we present focus on self-perceived learning gains and the identification of factors that could facilitate IPL in a time-limited format.

Both professions equally reported increased understanding of interprofessional roles and tasks through SIESTA. Similarly, both indicated a lower barrier for initiating interprofessional communication and exposing differences between professions was mentioned as important. Awareness for existing differences might be a first step towards better collaboration and communication. Importantly, as communication was facilitated, participants reported a sense of value for the other profession.

Improved self-perception of IP learning goals has been reported for different IPL activities, including interprofessional training wards [17, 46]. An improved understanding of mutual IP roles and an increased readiness to seek advice have been shown by others [23, 47].

Participants reported an at least moderate learning gain. The workplace-based nature of SIESTA was highlighted in focus group discussions, as was the aspect of a safe learning environment through peer learning.

Focus group discussions pointed towards too much theoretical input presenting an obstacle to effective learning. SIESTA was regarded as beneficial especially if there were practical things to do or show each other. Successful IP activities involving mainly practical aspects have been reported previously [20, 21]. Thus, interprofessional facilitators should emphasize and support procedure-related and practical topics (see below).

 Concerning self-reported learning gains, participants seemed to benefit from SIESTA even if they did not regard its content as “highly relevant”. This might point toward a more IP-centred view of learning gains. Different reports from IP projects around the world have emphasized the difference between profession-specific and interprofessional learning goals [10]. Thus, when assessing self-perception of learning gains, close attention should be paid to make this differentiation understandable to students.

Participants stated that 30 minutes was sufficient for a concise peer-led learning activity, if they completed pre-session preparation. The role of interprofessional facilitators was and should therefore be to remind participants of a planned session and encourage them to agree on a topic, which is one condition for successful IPL with time restriction.

Choosing topics with a practical side or a procedure to them should be emphasized. Facilitators can help by providing material such as inhalators or dressings. Generally, Participants mentioned facilitators as an important resource. Especially having both nursing and medical experts available during SIESTA was appreciated. Evaluation of IPL by facilitators has generally been positive in the past [19]. Although more time-consuming, facilitators found teaching in IP contexts rewarding [10]. It is essential for learners to have facilitators as IP role models during their IPL activities. Even if facilitators may not have had first-hand IPE opportunities, they should be inspired by their students’ role modelling [10].

### Strengths and limitations

Strengths of this study include the use of both quantitative and qualitative methods. Methodological triangulation was used to address our research questions and we were able to answer questions that arose from quantitative data by analysing qualitative data, too. For a study conducted in the context of one interprofessional training ward, the number of participants (*n* = 40) is acceptable and the response rate was near 100 %. 

One important limitation is the post-test only, single group design. While this holds true for many published IPE projects, future research should be designed to develop objective tools, specifically assess interprofessional skills, look at effects on a short- and long-term basis and ultimately include relevant patient and hospital outcomes [10]. Future studies should aim at an objective assessment of the IP learning progress. Methodologically, although questionnaires were based on established questionnaires, they have not been piloted or validated for the specific purpose of evaluating SIESTA. Combining questions from different questionnaires or changing them may influence validity. Therefore, semi-guided focus group discussions were used as a source of qualitative data aiming at internal validation of the findings from questionnaires. Concerning SIESTA as a single project, sessions were conducted in the context of an IP training ward. It is very likely that this has an influence on our data and should be taken into consideration. IP teams got to know each other during their daily work on the IP training ward and their perception of SIESTA might be influenced by that. Also, deriving SIESTA topics from patients’ clinical problems was facilitated in the context of the IP training ward placement. Although questionnaires were based on and adapted specifically to address SIESTA sessions, and semi-guided focus group discussions included questions dedicated to SIESTA, our data acquisition instruments also evaluated aspects of the overall IPAPAED project.

## Conclusion

Our data show that SIESTA sessions promoted self-perceived understanding of interprofessional roles and tasks and were a successful pilot for a time-limited, peer-led IPL activity. Communication and cooperation were key aspects to successful IPL. Using peer teaching methods, students felt in a safe learning environment and were eager to learn with, from and about each other. Learning fields involving a practical side were mentioned as particularly useful.

 Promoting short and easy-to-implement formats like SIESTA will hopefully convince faculty to enable many more students to benefit from IPL experiences on their way to becoming health care professionals.

## Supplementary Information

**Additional file 1.** SIESTA questionnaire. Full SIESTA questionnaire as it was handed to SIESTA participants. The original version of the questionnaire was in German. SF translated the questionnaire, with comments and corrections from all authors. All authors approved the final version of the translated questionnaire.

**Additional file 2.** Answers to SIESTA questionnaire items. Answers given by number of participants for the six SIESTA questionnaire items reported in this study, including percentage. P-values are provided for comparison between professions, using Mann-Whitney-U-test.

## Data Availability

All original data and questionnaires used are available from the corresponding author upon reasonable request.
