# Supplementary material for: SIESTA: a quick interprofessional learning activity fostering collaboration and communication between paediatric nursing trainees and medical students
Source: BMC Med Educ. 2021 Sep 6;21:475. doi: 10.1186/s12909-021-02880-9 (PMC8422752; doi:10.1186/s12909-021-02880-9)
Supplement: Supplementary file 1 — Additional file 1. SIESTA questionnaire. Full SIESTA questionnaire as it was handed to SIESTA participants. The original version of the questionnaire was in German. SF translated the questionnaire, with comments and corrections from all authors. All authors approved the final version of the translated questionnaire. [file 12909_2021_2880_MOESM1_ESM.docx]

**SIESTA Questionnaire**

1. I am…

| □ a trainee in paediatric nursing | □ a medical student |
| --- | --- |

1. Learning with and from the other profession during SIESTA…

|  | **strongly agree** | **agree** | **undecided** | **dis-agree** | **strongly disagree** |
| --- | --- | --- | --- | --- | --- |
|  | **1** | **2** | **3** | **4** | **5** |
| … contributed to a better understanding of the medical disorders discussed. |  |  |  |  |  |
| … contributed to a better communication of professional matters to parents/patients. |  |  |  |  |  |
| … contributed to a better understanding of the other profession’s tasks in patient care. |  |  |  |  |  |
| … made it more likely for me to seek advice from the other profession regarding patient care. |  |  |  |  |  |

1. The interprofessional collaboration of the SIESTA instructors was…

| excellent | good | fair | poor | very poor |
| --- | --- | --- | --- | --- |
| 1 | 2 | 3 | 4 | 5 |

1. Please rate the interprofessional exchange among the SIESTA participants.

| excellent | good | fair | poor | very poor |
| --- | --- | --- | --- | --- |
| 1 | 2 | 3 | 4 | 5 |

1. The contents discussed were highly relevant (e.g. for exams, professional work etc.).

| strongly agree | agree | undecided | disagree | strongly disagree |
| --- | --- | --- | --- | --- |
| 1 | 2 | 3 | 4 | 5 |

1. Please rate your learning gains through the SIESTA course.

| very high | high | medium | low | very low |
| --- | --- | --- | --- | --- |
| 1 | 2 | 3 | 4 | 5 |

1. Participating in the other profession’s handovers during SIESTA is a valuable experience.

| strongly agree | agree | undecided | disagree | strongly disagree |
| --- | --- | --- | --- | --- |
| 1 | 2 | 3 | 4 | 5 |

1. The duration of SIESTA (30 min) was…

| Far too long | Too long | Just right | Too short | Far too short |
| --- | --- | --- | --- | --- |
| 1 | 2 | 3 | 4 | 5 |

1. In your opinion, how often should SIESTA ideally take place during the two weeks placement on IPAPAED?

| More frequently | As offered | Less frequently |
| --- | --- | --- |
| 1 | 2 | 3 |

**Which** **further material or equipment would you have liked for SIESTA?**

|  |
| --- |

**What did you like regarding SIESTA?**

|  |
| --- |

**What could be improved?**

|  |
| --- |

**We look forward to your additional comments below!**

|  |
| --- |

**Thank you for your participation!**
