# Supplementary material for: SIESTA: a quick interprofessional learning activity fostering collaboration and communication between paediatric nursing trainees and medical students
Source: BMC Med Educ. 2021 Sep 6;21:475. doi: 10.1186/s12909-021-02880-9 (PMC8422752; doi:10.1186/s12909-021-02880-9)
Supplement: Supplementary file 2 — Additional file 2. Answers to SIESTA questionnaire items. Answers given by number of participants for the six SIESTA questionnaire items reported in this study, including percentage. P-values are provided for comparison between professions, using Mann-Whitney-U-test. [file 12909_2021_2880_MOESM2_ESM.docx]

Answers given by number of participants

| 1) Learning with and from the other profession during SIESTA contributed to a better understanding of the other profession’s tasks in patient care. | | | | | | | | | | | | | |
| --- | --- | --- | --- | --- | --- | --- | --- | --- | --- | --- | --- | --- | --- |
|  | | strongly agree | | agree | | undecided | | | disagree | | strongly disagree | | Total |
| NT (n, %) | | 18 (90%) | | 2 (10%) | | 0 (0%) | | | 0 (0%) | | 0 (0%) | | 20 (100%) |
| MS (n, %) | | 12 (60%) | | 8 (40%) | | 0 (0%) | | | 0 (0%) | | 0 (0%) | | 20 (100%) |
|  | | | | | | | | | | | | | |
| 2) Learning with and from the other profession during SIESTA made it more likely for me to seek advice from the other profession regarding patient care. | | | | | | | | | | | | | |
|  | | strongly agree | | agree | | undecided | | | disagree | | strongly disagree | | Total |
| NT (n, %) | | 13 (65%) | | 4 (20%) | | 3 (15%) | | | 0 (0%) | | 0 (0%) | | 20 (100%) |
| MS (n, %) | | 15 (75%) | | 3 (15%) | | 2 (10%) | | | 0 (0%) | | 0 (0%) | | 20 (100%) |
|  | | | | | | | | | | | | | |
| 3) Please rate the interprofessional exchange among the SIESTA participants. | | | | | | | | | | | | | |
|  | | excellent | | very good | | fair | | | poor | | very poor | | Total |
| NT (n, %) | | 12 (60%) | | 8 (40%) | | 0 (0%) | | | 0 (0%) | | 0 (0%) | | 20 (100%) |
| MS (n, %) | | 14 (70%) | | 6 (30%) | | 0 (0%) | | | 0 (0%) | | 0 (0%) | | 20 (100%) |
|  | | | | | | | | | | | | | |
| 4) The content discussed during SIESTA was highly relevant (e.g. for exams, professional work etc.). | | | | | | | | | | | | | |
|  | strongly agree | | agree | | undecided | | | disagree | | strongly disagree | | Total | |
| NT | 7 (36%) | | 8 (42%) | | 2 (11%) | | | 2 (11%) | | 0 (0%) | | 19 (100%) | |
| MS | 9 (45%) | | 11 (55%) | | 0 (0%) | | | 0 (0%) | | 0 (0%) | | 20 (100%) | |
|  | | | | | | | | | | | | | |
| 5) Please rate your learning gains through the SIESTA course. | | | | | | | | | | | | | |
|  | very high | | high | | moderate | | | low | | very low | | Total | |
| NT | 6 (30%) | | 9 (45%) | | 5 (25%) | | | 0 (0%) | | 0 (0%) | | 20 (100%) | |
| MS | 4 (20%) | | 11 (55%) | | 5 (25%) | | | 0 (0%) | | 0 (0%) | | 20 (100%) | |
|  | | | | | | | | | | | | | |
| 6) Please rate the duration of SIESTA (30 min). | | | | | | | | | | | | | |
|  | far too long | | too long | | just right | | too short | | | Far too short | | Total | |
| NT | 0 (0%) | | 1 (5%) | | 4 (20%) | | 11 (55%) | | | 4 (20%) | | 20 (100%) | |
| MS | 0 (0%) | | 0 (0%) | | 9 (45%) | | 9 (45%) | | | 2 (10%) | | 20 (100%) | |

Questions 1) and 2): p = 0.48 between professions

Question 3): p = 0.74 between professions

Question 4): p = 0.32
Question 5): p = 0.82

Question 6): p = 0.21

NT = paediatric nursing trainee, MS = medical student
